# Supplementary material for: From sequence to enzyme mechanism using multi-label machine learning
Source: BMC Bioinformatics. 2014 May 19;15:150. doi: 10.1186/1471-2105-15-150 (PMC4229970; doi:10.1186/1471-2105-15-150)
Supplement: Additional file 2 — Java code of ml2db. Additional file ml2db_code.tar.gz contains the Java source code to run the multi-label machine learning experiments and save the results to database. The code’s Javadoc is included. [file 1471-2105-15-150-S2.zip › additional file 2/ml2db/utils/doc/uk/ac/standrews/utils/main/database/Table.html]

Table


JavaScript is disabled on your browser.


- Overview
- Package
- Class
- Use
- Tree
- Deprecated
- Index
- Help

- Prev Class
- Next Class

- Frames
- No Frames

- All Classes

- Summary:
- Nested |
- Field |
- Constr |
- Method

- Detail:
- Field |
- Constr |
- Method


uk.ac.standrews.utils.main.database

## Class Table

- java.lang.Object
- - uk.ac.standrews.utils.main.database.Table

- ---

    

  ```
  public class Table
  extends java.lang.Object
  ```

  An SQL database table definition (table name + columns)

  Version:
  :   10 Jun 2010

  Author:
  :   Luna De Ferrari luna.deferrari-at-ed.ac.uk

- - ### Field Summary

    Fields

    | Modifier and Type | Field and Description |
    | --- | --- |
    | `TableWriter` | `m_writer` the table writer (to write data to this table) |
  - ### Constructor Summary

    Constructors

    Constructor and Description

    `Table(java.lang.String tableName,
    DbManager dbManager)`

    Construct a table read-only.

    `Table(java.lang.String tableName,
    java.util.Vector<TableColumn> columns,
    DbManager dbManager)`

    Construct a table for writing to: the columns definitions (name, data type) must be known.
  - ### Method Summary

    Methods

    | Modifier and Type | Method and Description |
    | --- | --- |
    | `java.util.Vector<java.lang.String>` | `getColumnNames()` Gets column names for the table |
    | `java.util.Vector<TableColumn>` | `getColumns()` Get the `TableColumn` list |
    | `DbManager` | `getDbManager()` public int saveRowToTable(TableRow row) { // write row to table TableWriter writer = this.getTableWriter(); int resultId = writer.insertReturnsKey(row); if (m\_debug ) { System.out.println("Written row with id: " + resultId + " to table " + this.getTableName() + " database " + this.getDbManager().getDatabaseName() + " - " + this.getClass().getName()); } return resultId; } |
    | `TableCreator` | `getTableCreator()` |
    | `java.lang.String` | `getTableName()` Get the table name |
    | `TableReader` | `getTableReader()` |
    | `TableRow` | `getTableRow()` |
    | `TableWriter` | `getTableWriter()` |
    | `boolean` | `hasKey()` Gets the "has primary key" status for the table |
    | `void` | `initialise(java.lang.String tableName, DbManager dbManager)` |
    | `void` | `initialise(java.lang.String tableName, DbManager dbManager, java.util.Vector<TableColumn> columns)` |
    | `java.lang.String` | `name()` |
    | `int` | `saveRow(java.util.TreeMap<java.lang.String,java.lang.String> map)` |

    - ### Methods inherited from class java.lang.Object

      `equals, getClass, hashCode, notify, notifyAll, toString, wait, wait, wait`

- - ### Field Detail


    - #### m\_writer

      ```
      public TableWriter m_writer
      ```

      the table writer (to write data to this table)
  - ### Constructor Detail


    - #### Table

      ```
      public Table(java.lang.String tableName,
           java.util.Vector<TableColumn> columns,
           DbManager dbManager)
      ```

      Construct a table for writing to: the columns definitions (name, data type) must be known.

      Parameters:
      :   `tableName` -
      :   `columns` -
      :   `dbManager` -


    - #### Table

      ```
      public Table(java.lang.String tableName,
           DbManager dbManager)
      ```

      Construct a table read-only. The full column definitions (column name, type, primary keys etc.)
      are not needed as the table will not be created by this Table object.

      Parameters:
      :   `tableName` -
      :   `columns` -
      :   `dbManager` -
  - ### Method Detail


    - #### getColumnNames

      ```
      public java.util.Vector<java.lang.String> getColumnNames()
      ```

      Gets column names for the table

      Returns:
      :   the column names


    - #### getColumns

      ```
      public java.util.Vector<TableColumn> getColumns()
      ```

      Get the `TableColumn` list

      Returns:
      :   the `TableColumn` list


    - #### getTableName

      ```
      public java.lang.String getTableName()
      ```

      Get the table name

      Returns:
      :   a string with the table name


    - #### hasKey

      ```
      public boolean hasKey()
      ```

      Gets the "has primary key" status for the table


    - #### initialise

      ```
      public void initialise(java.lang.String tableName,
                    DbManager dbManager)
      ```


    - #### initialise

      ```
      public void initialise(java.lang.String tableName,
                    DbManager dbManager,
                    java.util.Vector<TableColumn> columns)
      ```


    - #### getTableRow

      ```
      public TableRow getTableRow()
      ```


    - #### getTableReader

      ```
      public TableReader getTableReader()
      ```


    - #### getTableWriter

      ```
      public TableWriter getTableWriter()
      ```


    - #### getTableCreator

      ```
      public TableCreator getTableCreator()
      ```


    - #### name

      ```
      public java.lang.String name()
      ```

      Returns:


    - #### getDbManager

      ```
      public DbManager getDbManager()
      ```

      public int saveRowToTable(TableRow row) {
      // write row to table
      TableWriter writer = this.getTableWriter();
      int resultId = writer.insertReturnsKey(row);
      if (m\_debug ) {
      System.out.println("Written row with id: "
      + resultId
      + " to table "
      + this.getTableName()
      + " database "
      + this.getDbManager().getDatabaseName() + " - "
      + this.getClass().getName());
      }
      return resultId;
      }


    - #### saveRow

      ```
      public int saveRow(java.util.TreeMap<java.lang.String,java.lang.String> map)
      ```

      Parameters:
      :   `map` - a map of table column name to value

      Returns:
      :   the identifier of the table row just inserted


- Overview
- Package
- Class
- Use
- Tree
- Deprecated
- Index
- Help

- Prev Class
- Next Class

- Frames
- No Frames

- All Classes

- Summary:
- Nested |
- Field |
- Constr |
- Method

- Detail:
- Field |
- Constr |
- Method
